# A power law distribution of metabolite abundance levels in mice regardless of the time and spatial scale of analysis

**DOI:** 10.1038/s41598-018-28667-5

**Published:** 2018-07-09

**Authors:** Shumpei Sato, Makoto Horikawa, Takeshi Kondo, Tomohito Sato, Mitsutoshi Setou

**Affiliations:** 1 Department of Cellular and Molecular Anatomy, Hamamatsu University School of Medicine, 1-20-1 Handayama Higashi-ku, Hamamatsu, Shizuoka, 431-3192 Japan; 2 International Mass Imaging Center, Hamamatsu University School of Medicine, 1-20-1 Handayama Higashi-ku, Hamamatsu, Shizuoka, 431-3192 Japan; 3 Preeminent Medical Photonics Education & Research Center, 1-20-1 Handayama, Higashi-ku, Hamamatsu, Shizuoka, 431-3192 Japan; 4 Department of Anatomy, The University of Hong Kong, 6/F, William MW Mong Block 21 Sassoon Road, Pokfulam, Hong Kong SAR, China

## Abstract

Biomolecule abundance levels change with the environment and enable a living system to adapt to the new conditions. Although, the living system maintains at least some characteristics, e.g. homeostasis. One of the characteristics maintained by a living system is a power law distribution of biomolecule abundance levels. Previous studies have pointed to a universal characteristic of biochemical reaction networks, with data obtained from lysates of multiple cells. As a result, the spatial scale of the data related to the power law distribution of biomolecule abundance levels is not clear. In this study, we researched the scaling law of metabolites in mouse tissue with a spatial scale of quantification that was changed stepwise between a whole-tissue section and a single-point analysis (25 μm). As a result, metabolites in mouse tissues were found to follow the power law distribution independently of the spatial scale of analysis. Additionally, we tested the temporal changes by comparing data from younger and older mice. Both followed similar power law distributions, indicating that metabolite composition is not diversified by aging to disrupt the power law distribution. The power law distribution of metabolite abundance is thus a robust characteristic of a living system regardless of time and space.

## Introduction

Biomolecule abundance levels change depending on the environmental conditions and enable a living system to adapt to the new conditions^1^. Many omics data analyses have revealed that molecules are upregulated or downregulated under different conditions, such as gene mutations or changes in food supply. On the other hand, a living system also has some characteristics that are maintained under all circumstances, e.g. homeostasis. In addition, a living system is subject to various limitations under which it has evolved. In recent years, omics data analysis has progressively developed^2–4^. They have the potential to reveal the maintained characteristics of a living system and the power law is one of them.

Power law distribution is a kind of data distribution that is approximated by the function $y=c{x}^{-k}$. Many phenomena have been reported to follow the power law distribution; for example, word frequency in texts, number of references in publications, earthquake frequencies for various regions, and so on^5,6^. Moreover, power law distribution analyses have also contributed to biological research^7–10^. Furthermore, biomolecule abundance and pathway networks have been reported to obey the power law in a living system^11–15^. Highly advanced techniques previously revealed that a wide range of organisms, from bacteria to humans, follow a power law distribution in their mRNA abundance levels^12,13^. Additionally, the power law distribution of biomolecule abundance was previously reported for the proteome and metabolome after the comprehensive analysis of mass spectrometry omics data^14,15^. The results of these studies suggest that the power law is a universal feature of biomolecule abundance levels in a living system; many biomolecules are present at low abundance levels and only a few at very high levels. However, in these studies, the data used to investigate the power law distribution was a result of analyses carried out on multiple cell lysates, such as from a specific organ, thereby leading to the loss of information on spatial distribution. The tissues of a multicellular organism have a specific structure within which biomolecules are distributed spatially. It remains to be seen whether the spatial distribution of biomolecules is related to the current understanding of the power law distribution of biomolecule abundance.

In this study, we analyzed data using a specific kind of mass spectrometry that collects data on molecule abundance with spatial location information, matrix assisted laser desorption/ionization imaging mass spectrometry (MALDI IMS). This method can perform both comprehensive analyses of molecules by mass spectrometry and imaging in a tissue slice^16,17^. This method involves coating tissue samples with a matrix as an ionization inducer and performs punctiform mass spectrometry (uniformly spaced points to be analyzed for imaging). The averaged mass spectrum of an arbitrary area is obtained by calculating the average of the mass spectra of each measuring point. Each peak intensity of the mass spectrum indicates the peak’s molecule abundance defined by its *m/z*. In this study, we mainly targeted metabolites, including lipids (phosphatidic acid, phosphatidylethanolamine, phosphatidylserine, phosphatidylglycerol, phosphatidylinositol, and sulfatide can be detected empirically in a method we used that uses 9-aminoacridine (9AA) as a matrix^18^). We then tested whether the peak intensities in an averaged mass spectrum of mouse tissues followed a power law distribution in a region of any size, in other words, according to changes in the number of measuring points. This analysis helped us evaluate the spatial scale that the power law distribution of biomolecule abundance levels can be observed.

Furthermore, in the following step, we tested for changes over time by observing the peak intensity distribution of samples taken from aged mice. Generally, aging is thought to be a complex process that progresses in several ways^19–21^. This suggests that the metabolite composition in each cell may become increasingly disordered with age. Increasing the variation of the metabolite composition in each cell gives rise to the disruption of the power law distribution in tissues. As a result, we expected the power law distribution to be disrupted from local regions in the tissue sample because variations in cellular metabolite composition may increase with age because of the decrease of molecular fidelity^20^. In addition, the power law distribution of the averaged metabolite abundance levels was expected to be close to the uniform distribution of that average, and it is known that several characteristics of a living system are disrupted with age. Hence, we analyzed the IMS data to clarify the change in the power law distribution of metabolite abundance levels over time and space.

## Results

**Figure 1:**
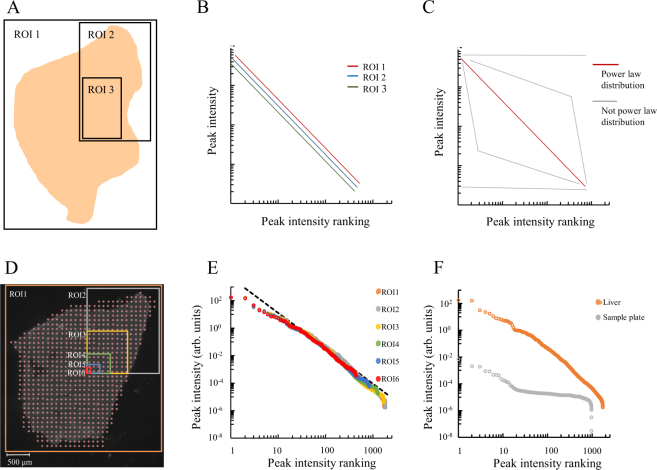
A rank-ordered distribution of peak intensities of various sized ROIs in a mouse liver tissue slice. The images and actual analysis of mouse tissue, regions of interest (ROIs), and their peak intensity rank-ordered distribution. (**A**) Mouse tissue and ROIs, with different sizes. (**B**) Peak intensity rank-ordered distribution of each ROI in panel A. The vertical axis shows the peak intensity, while the horizontal axis shows the rank of corresponding peak intensity. Both axes have a logarithmic scale. If the power law distribution is observed regardless of tissue structures, distributions of any ROI should match the power law distribution. (**C**) Distributions as the power law distribution changes along with changes in ROI size. The slope and curvature of a distribution are criteria for identifying the power law distribution. (**D**) Image of a mouse liver analyzed by MALDI IMS. Measuring points (pink dots) and the analyzed regions (ROIs) for acquiring the averaged mass spectrum. (**E**) The peak intensity rank-ordered distributions of each ROI in panel D. The colors of distributions is consistent with the colors of ROIs shown in panel D. The dashed line is a reference to the power law distribution. (**F**) The peak intensity rank-ordered distribution of ROI 1 in panel D and of a sample plate only.

The aim of this study was to evaluate whether the ordered distribution of peak intensities constructed from the averaged mass spectrum of a region of interest (ROI) always follows the power law distribution even when the size of an ROI is reduced (Fig. 1A). When the log-log plot of a rank-ordered distribution of peak intensities is a straight line, the distribution is known as “a power law distribution” (Fig. 1B). Conversely, when the distribution is not a straight line or clearly shows a zero slope, the distribution is labeled as “not a power law distribution” (Fig. 1C). First, we investigated the peak intensity rank-ordered distribution in the averaged mass spectrum of each region within a mouse liver tissue slice. The averaged mass spectrum is the average of the mass spectra of each measuring point. The mass spectrum of one measuring point (Fig. 1D, pink dots) is a cumulative spectrum of 200 spectra produced by the molecules ionized by a laser shot 200 times with a beam diameter of approximately 25 μm. The size of the analyzed regions (Fig. 1D, ROIs) was reduced from a whole tissue section to a single point, arbitrarily and stepwise (approximately quartered). Numbers of measuring point were 710, 200, 81, 20, 6, and 1 in ROI 1, 2, 3, 4, 5, and 6 respectively. Then, when the peak intensities and their rank were plotted according to the averaged mass spectrum data, the log-log plot of peak intensity rank-ordered distributions were found to be linear distributions with similar slopes in all ROIs and were designated as the power law distribution (Fig. 1E). The maximum values of peak intensities were similar for all ROI. The peak number was found to decrease along with the reduction in the number of measuring points. Although the peak number in the whole-tissue analysis (ROI 1) was 1727, it was 433 in the one-point analysis (ROI 6). On the other hand, the peak intensity rank-ordered distribution of the sample plate measurements without tissue slice samples did not follow the power law distribution, and the maximum values of peak intensities were relatively low (Fig. 1F, Liver ROI 1: 183.117 arbitrary units, sample plate: 0.003 arbitrary units). In Fig. 1, the peak intensity rank-ordered distribution showed the power law distribution from whole-tissue to one-point analyses.

**Figure 2:**
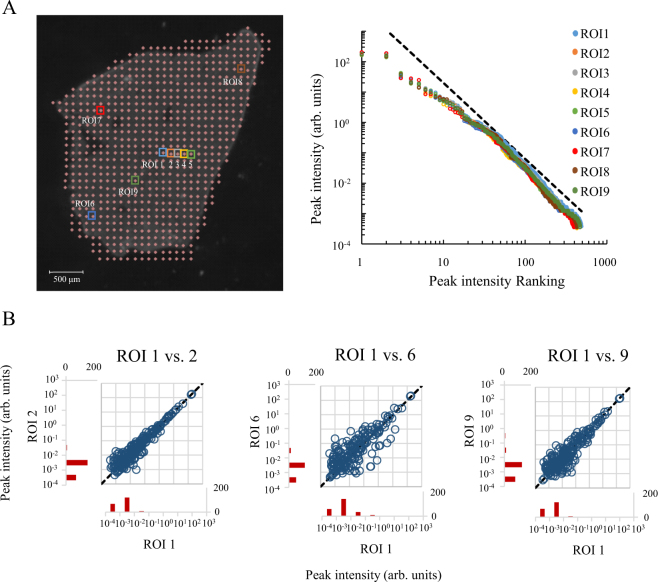
Comparisons of the one-point analysis data from a mouse liver tissue section. (**A**) Locations of each one-point analysis data (ROIs) in a mouse liver section (left panel) and their peak intensity rank-ordered distributions. The colors of distributions are consistent with the colors of ROIs shown in the left panel. The dashed line is a reference to the power law distribution. (**B**) A scatter plot of each peak intensity observed in both one-point analysis comparison data and in the histogram of peak numbers binned by the order of intensity observed on only one side. The dashed line refers to unchanged peaks.

Next, we investigated localization of the analysis point following the power law distribution on a tissue slice. For example, when only one measuring point shows strong intensity and follows the power law distribution, it should overwhelm the other distributions, and the peak intensity rank-ordered distributions should display very different distributions depending on whether the ROI includes the one-point analysis data. Thus, we investigated whether the power law distribution for one-point analyses is a general rule by plotting the peak intensity rank-ordered distributions of mass spectra obtained by some of the one-point analyses data obtained from a mouse liver slice (Fig. 2; each ROI contained only a single measuring point.). The one-point analysis data were chosen from adjacent regions of interest (ROIs; ROI 1–5), distant ROIs (ROI 6–8), and a region of the vascular system that is anatomically different from ROI 1–8 (ROI 9). As a result, all peak intensity rank-ordered distributions obtained from the one-point analyses followed the power law distribution. The peak number and range of peak intensities were similar among all one-point analyses.

The power law distribution was observed for all one-point analyses (Fig. 2A). Incidentally, the peaks constituting these distributions could be either similar or different between measuring points. Although certain molecular species show specific localization in tissues, e.g. neurotransmitters, certain molecular species are distributed uniformly, e.g. DNA. To evaluate the effect that specific localization of the molecules constituting the power law distribution in our analysis has on peak construction, we compared the intensities of the same *m/z* peaks between some of the one-point analysis results. Using these results, we also evaluated whether the power law distribution was maintained regardless of the peak compositions being different or similar. Peaks that were commonly detected in both ROI 1 and ROI 2 showed similar intensities (Fig. 2A), while especially low-intensity peaks showed similar variability (Fig. 2B, scatter plot). The maximum value of the difference between peak intensities in all comparisons is approximately 100-fold. The peaks were not skewed to one side; they were spread uniformly between ROIs. The percentage of commonly detected peaks between both ROIs was approximately 50%. The remaining peaks were detected in only one-side of an ROI. In addition, these uncommon peaks had a relatively low intensity. Similarly, ROI 1 and 6, and ROI 1 and 9 in Fig. 2A were also compared and showed certain similarity and variability of commonly detected peaks, and a similar shape to the histogram of low-intensity, not commonly detected peaks, as in the comparison of ROIs 1 and 2. Thus, the variability of peak intensity was found within a certain range. Peaks were distributed with different intensities as shown in Fig. 2B, and their variability was uniform between ROIs.

**Figure 3:**
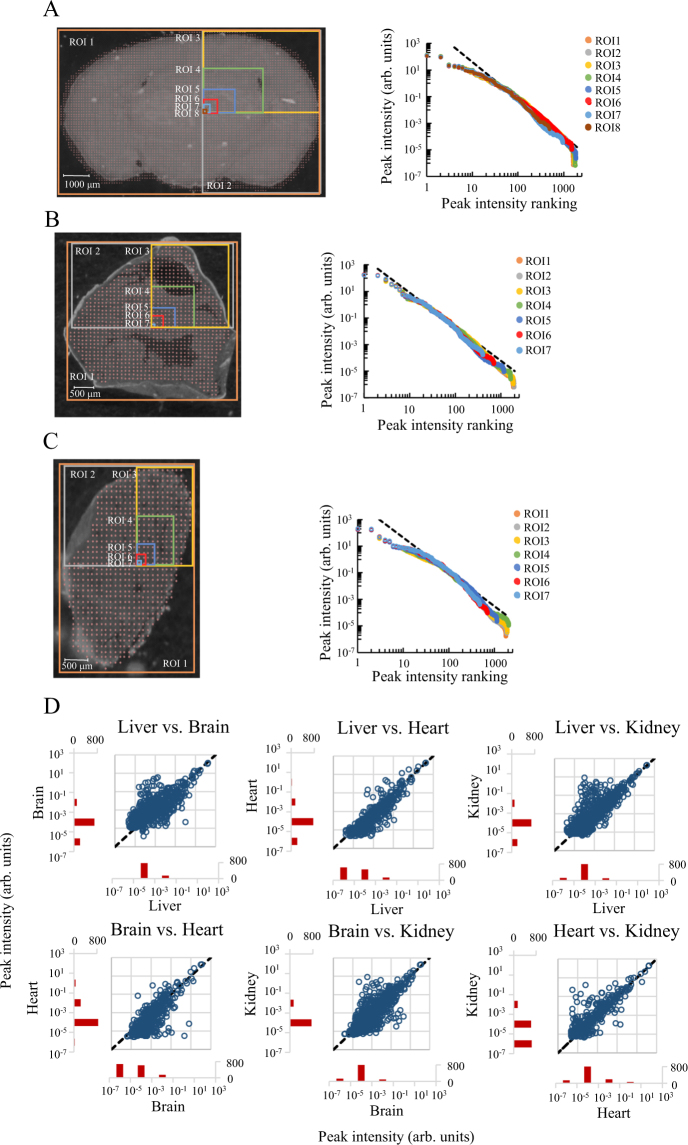
Rank-ordered distribution of peak intensities in mouse brain, heart, and kidney tissue slices in ROIs of various sizes and comparisons of peak composition between organs. Images of tissue slices of the mouse (**A**) brain, (**B**) heart, and (**C**) kidney analyzed by MALDI IMS (left panels) and their peak intensity rank-ordered distributions (right panels). The colors of distributions is consistent with colors of ROIs shown in the left panel. The dashed line is a reference to the power law distribution. (**D**) A scatter plot of each peak intensity observed in an averaged spectrum of each whole tissue section (ROI 1 of Fig. 3A–C) and a histogram of the peak numbers binned by the order of intensity observed on only one side tissue. The dashed line refers to unchanged peaks.

The above results were obtained from mouse liver tissue; therefore, the observed feature, i.e. that the power law distribution of the peak intensity rank-ordered distribution appears to be independent of the spatial region size of quantification, may be true only for the liver. To test this possibility, we analyzed the distributions in the brain (Fig. 3A), heart (Fig. 3B), and kidney (Fig. 3C) tissues of mouse, with changes in spatial region size of quantification similar to those applied in the liver tissue samples. In brain tissue, numbers of measuring point were 5371, 2589, 1389, 475, 143, 30, 9 and 1 in ROI 1, 2, 3, 4, 5, 6, 7, and 8 respectively, in heart tissue, 1068, 545, 305, 121, 36, 9, and 1 in ROI 1, 2, 3, 4, 5, 6, and 7 respectively, and in kidney tissue, 706, 365, 213, 63, 16, 4, and 1 in ROI 1, 2, 3, 4, 5, 6 and 7 respectively. As a result, in these tissues, the power law distributions were found to be independent of the size of the analyzed spatial region, in agreement with the liver tissue results. The maximum values of the peak intensities were similar between ROIs in one organ and between multiple organs. In addition, a decrease in the number of peaks was observed along with a reduction in the analyzed spatial region size, in line with the results obtained for the liver slices. Thus, the power law distributions, independent of the analyzed spatial region sizes, were also independent of the organs. The peak compositions between different tissues were plotted as in Fig. 2B (Fig. 3D). The averaged spectra from whole tissues were used for this plot. All combinations were tested (liver, brain, heart, and kidney). The number of common (scatter plot) peaks was approximately 600 and that of uncommon peaks (histogram) was approximately 1000. Comparing with single measuring point analyses, the numbers of common peak was increased 3-fold with increase of the number of measuring points. As shown in Fig. 2B, all the pairs showed a similar intensities, and low-intensity peaks showed a similar variability (Fig. 3D). The maximum value of difference of peak intensities in all comparisons was found to be approximately 1 × 10^6^-fold. This is 1 × 10^4^-fold larger than that of Fig. 2B.

**Figure 4:**
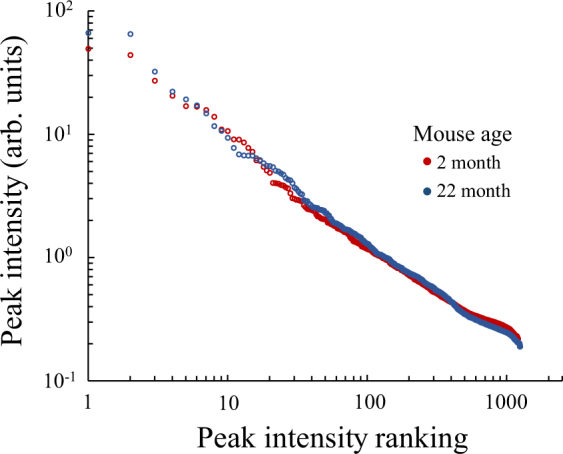
A rank-ordered distribution of peak intensities of mouse livers aged 2 and 22 months. Peak intensity rank-ordered distributions of averaged mass spectrum of liver slice imaging data from mice aged 2 and 22 months.

Following the test for spatial differences in IMS analysis, we then tested against changes in time. For this, we used the IMS data from liver tissues of mice aged 2 and 22 months. The power law distributions were obtained from the peak intensities of the averaged mass spectrum of whole liver tissue slices of both aged mouse livers and compared. As a result, all mice were found to follow similar power law distributions (Fig. 4). In addition, the maximum and minimum peak intensities, and the peak numbers, were found to be similar in both samples.

## Discussion

With MALDI IMS for the analysis of negative ions using a 9AA matrix, certain nucleotides^22^, oligonucleotides^22^, deoxynucleotides^23^, amino acids^24^, peptides^24^, sugars^25^, glycosides^26^, and lipids^18^ have been previously reported as detected components, and lipids and low-molecular-weight metabolites as the main components^18,27,28^. As a result, we could observe only a part of the metabolome in this study. However, our IMS data may reflect at least part of the real distribution of metabolite abundance levels since some of the IMS data, taken from biological samples following the power law distribution^13,14^, supports that the amount of biomolecules in the living system obeys the power law. In the MALDI IMS data of mouse liver slices, the peak intensity rank-ordered distribution of the averaged mass spectrum followed the power law distribution independently from the number of measuring points (Fig. 1). This indicates that the metabolite abundance levels of the mouse liver obey the power law regardless of the size of the region being analyzed. However, it is important to note that we examined a region of size larger than a single cell because the diameter of one measuring point is approximately 25 μm. Liver cells, which occupy 80% of the liver volume, also have a diameter of approximately 25 μm^29,30^. Furthermore, we chose the points for the one-point analyses (Fig. 1A, ROI 9) to include the vascular system, which contains other types of cells, because most of the other measuring points (Fig. 2A, ROI 1–8) probably consisted mostly of hepatocytes. Their metabolite abundance levels also followed a power law distribution similar to the other one-point analyses. Furthermore, the metabolite composition of each measuring point was different because the peak compositions of the measuring points were different to a certain extent (Fig. 2B). Therefore, the power law distribution of metabolite abundance levels may be a feature that is observed in any cell independently of its metabolite composition. The peaks not commonly detected in the data comparisons were low-intensity peaks (Fig. 2B histogram); it seems that the abundance of these metabolites is close to the detection limit. Additionally, the histogram shape and peak for these uncommon peaks were similar in comparison; therefore, the peak composition and variability of the low-abundance metabolites may also be similar between measuring points.

Liver tissue is comprised of a hexagonal and lobular structure formed by hepatocytes and a central vein^29,31^. As a result, it is assumed that the composition of the cells is similar and corresponds to changes in size of the analyzed spatial region. The brain, heart, and kidneys have a relatively low spatial homogeneity of structure when compared to the liver. Despite this, in these tissues, the power law distribution of metabolite abundance levels was still observed (Fig. 3A–C). This finding suggests that the power law distribution is detected independently of the tissue structure. Additionally, in terms of the compositions between organs, the shape of the scatter plot was found to be spread equally from the center line of the plot (Fig. 3D). Bow-tie architecture permits possible extreme complexity throughout metabolism^32^. The modular architectures like that have a potential to generate the power law distribution and can explain why the power law distribution was maintained in all observations of this study. The power law distribution can be observed in an analyzed region of size larger than the organ level. In addition, scatter plots tend to spread wider than that of the comparison between single point analyses in Fig. 2B. These results indicate that the metabolite abundance was more different between cells in different organs than between cells in the same tissue. That is reasonable and also supports that our results reflect a part of real abundance of metabolites.

In this study, we demonstrated that the metabolite abundance levels of various mouse tissues obey the power law, whenever the metabolites present in any region of a tissue larger than 25 μm in diameter are quantified. We expected when diversity of cellular biomolecule compositions are increased, the power law distribution generated from the tissue is collapsed. However, the distributions were maintained and the peak intensities between measuring points were found to be correlated (Fig. 2B). The similar compositions of biomolecule abundance levels among cells may also be a reason for the maintenance of the power law distribution in any spatial scale of analysis. Similar result of bacterial study of metabolite^33^ supports this.

The molecular fidelity is decreased with age even if the cell has a maintenance system because of the second law of thermodynamics^20^. In addition, lipid metabolite abundance often shows large changes between young and old age^34,35^. Because of these reasons, we expected the largest difference of distribution in comparison of two points, young and old state of cells. Despite this, we found that the power law distributions were similar between younger and older mice (Fig. 4). Therefore, the components determining the metabolite reaction network may be one of the robust components of a living system that remained unaffected by aging. Since an increase in cell variation is expected to disrupt the power law distribution, aging was noted to not affect the increase in the variation of the cell metabolite composition enough to disrupt the power law distribution.

The mechanism behind the generation of a network that obeys the power law distribution can be explained by the preferential attachment principal: a vertex with a great amount of nodes has a higher probability of stochastically gaining a new node^36^. Zhu *et al*.^33^ indicated that the preferential attachment principal is the basis for the evolution of the metabolite network by showing that a more abundant hydrophilic metabolite has a higher probability to interconvert to a new metabolite for evolutionary expansion of metabolite network. Therefore, the metabolite pathway and abundance composition obey the power law. The lipid metabolite network expands from some basic species of lipid to the next. Therefore, in the expansion of the hydrophobic metabolite network with evolution of oxygen utilization^37^, the preferential attachment principal might work for lipid metabolite network in a similar way as for the whole metabolite network. Indeed, our results showed that the power law distribution of metabolite abundance levels was constructed mainly by lipids.

A scale free network (which obeys the power law distribution) is robust against error^11^. In this study, our results showed that the power law distribution is found regardless of spatial scales. This indicates that the reaction network structure of metabolites will be maintained even when the metabolite compositions are changed over a wide area of the body. Because age and mortality are positively correlated mostly in mammals^38^, it can be thought that aging decreases robustness to survive. Therefore, we expected to find a decrease in the robustness of the metabolite network in aged mice because of the presence of disordered cells in the body^19–21^. However, we found that the power law distribution was similar in both young and aged mice. As such, aging may not disrupt the network structure of the cell, but relatively subtle changes in metabolites could be a significant sign of aging. These changes can trigger cell death and eventually the death of the living system as a multicellular organism.

In conclusion, the power law distribution of metabolite abundance levels is a robust characteristic whose analysis is not affected by time (during the living systems’ lifespan) or space (from the cellular to tissue level).

## Materials and Methods

### Animals and chemicals

C57BL/6 J male mice aged 8 weeks were purchased from Japan SLC (Hamamatsu, Japan). All animal experiments were approved by the Animal Care and Use Committee of the Hamamatsu University School of Medicine and were carried out in accordance with the approved guidelines. Human angiotensin II and bradykinin, used as calibration standard peptides, were purchased from Sigma-Aldrich Japan (Meguro, Japan). 9AA, used as a matrix, was purchased from Merck Millipore (Darmstadt, Germany).

### Sample preparation

Three C57BL/6 J male mice were analyzed in this study. The brain, kidneys, heart, and liver from mice aged 2 months and the liver from the mouse aged 22 months were excised after euthanasia by cervical dislocation, and subsequently frozen with powdered dry ice. The frozen tissues were sectioned (10 μm) at −20 °C using a Cryostat (CM1950, Leica). For each evaluation, a set of tissue slices was mounted on an indium tin oxide-coated glass slide (100 Ω, Matsunami). The sample slides were sealed with silica gel and stored at −80 °C. After one day, the slides were transferred to a room temperature environment (approximately 25 °C) and then opened for matrix coating. The matrix was applied to slides as a layer of 1.0 μm-thick by sublimation using iMLayer (Shimadzu).

### MALDI IMS analysis

All samples, except those for the comparison of 2 and 22 month-old mice, were studied on an ultraflex II (Bruker Daltonics) with an *m/z* range of 50–1050, laser diameter at the smallest value (~25 μm), laser spot raster at 100 μm, and laser shot count of 200. For the external calibration of time of flight (TOF), 9AA ([M-H]^−^, *m/z* 193.1), a human bradykinin fragment (amino acid residues 1–7, [M-H]^−^, *m/z* 755.4), and human angiotensin II ([M-H]^−^, *m/z* 1044.5) were used. The comparison of 2 and 22 month-old mice was performed on a rapifleX (Bruker Daltonics) with an *m/z* range of 50–1050, laser diameter at the smallest value (~5 μm), 1 measuring point diameter of 25 μm, laser spot raster at 100 μm, and laser shot count of 200. The chemicals used for the external calibration for the rapifleX analysis were the same (9AA, bradykinin fragment, and angiotensin II) as used for the ultraflex II analyses.

### Data analysis

We acquired mass spectrum data from each measuring point in tissue samples by MALDI IMS analyses. A photo image of a tissue section and positions of measuring points were adjusted, and then ROIs were encircled on the image. The first ROI contained all measuring points and the size of ROI series was stepwisely reduced with similar ratio. The ROI size was reduced to reaching a single measuring point. The mass spectrum of each ROI was calculated as the mean spectrum of mass spectra of all measuring points in the ROI. The mass spectrum was exported from the flexImaging software (Bruker Daltonics) as numerical data, whose intensity on each *m/z* was divided equally by the 8000-point scale among the spectrum data of *m/z* 50–1050. Peaks were sampled and ordered by their intensities and ranked with an integer. For sampling of the same peaks, the *m/z* of each peak was rounded off to one decimal place, and peaks with the same rounded *m/z* value were assumed to be identical.

Regression for the power law distribution was derived from the linear regression of the logarithmically transformed data using the ordinary least square method, and the regression line was log-transformed to adapt it to a log-log plot. Some peaks were excluded to avoid a finite size effect from peaks close to the detection limit. The excluded peaks were determined by the smallest peak number of an ROI of each analyzed organ, and the peaks with a rank bigger than that criterion were excluded from the regression calculation.
